# Prediction for late-onset sepsis in preterm infants based on data from East China

**DOI:** 10.3389/fped.2022.924014

**Published:** 2022-09-14

**Authors:** Xianghua Shuai, Xiaoxia Li, Yiling Wu

**Affiliations:** Department of Neonatology, Affiliated Hangzhou First People's Hospital, Zhejiang University School of Medicine, Hangzhou, China

**Keywords:** sepsis, late-onset, prediction, preterm infants, East China

## Abstract

**Aim:**

To construct a prediction model based on the data of premature infants and to apply the data in our study as external validation to the prediction model proposed by Yuejun Huang et al. to evaluate the predictive ability of both models.

**Methods:**

In total, 397 premature infants were randomly divided into the training set (*n* = 278) and the testing set (*n* = 119). Univariate and multivariate logistic analyses were applied to identify potential predictors, and the prediction model was constructed based on the predictors. The area under the curve (AUC) value, the receiver operator characteristic (ROC) curves, and the calibration curves were used to evaluate the predictive performances of prediction models. The data in our study were used in the prediction model proposed by Yuejun Huang et al. as external validation.

**Results:**

In the current study, endotracheal intubation [odds ratio (*OR*) = 10.553, 95% confidence interval (*CI*): 4.959–22.458], mechanical ventilation (*OR* = 10.243, 95% *CI*: 4.811–21.806), asphyxia (*OR* = 2.614, 95% *CI*: 1.536–4.447), and antibiotics use (*OR* = 3.362, 95% *CI*: 1.454–7.775) were risk factors for late-onset sepsis in preterm infants. The higher birth weight of infants (*OR* = 0.312, 95% *CI*: 0.165–0.588) and gestational age were protective factors for late-onset sepsis in preterm infants. The training set was applied for the construction of the models, and the testing set was used to test the diagnostic efficiency of the model. The AUC values of the prediction model were 0.760 in the training set and 0.796 in the testing set.

**Conclusion:**

The prediction model showed a good predictive ability for late-onset sepsis in preterm infants.

## Introduction

Neonatal sepsis is a clinical syndrome caused by pathogenic bacteria, and can invade the blood circulation, affect the growth, and attack the reproduction system of neonates during the 1st month of life, which is characterized by hemodynamic changes and other systemic symptoms of infection (1). Neonatal sepsis may result in organ failure and tissue damage, which is a vital reason for neurocognitive sequelae and neonatal mortality (2, 3). According to the age of onset, neonatal sepsis is divided into early-onset sepsis and late-onset sepsis. Early-onset sepsis reflects transplacental or, more frequently, ascending infections from the maternal genital tract, whereas late-onset sepsis is associated with the postnatal nosocomial or community environment, with the peak incidence reported to be between the 10th and 22nd day of life (4). Patients with late-onset sepsis have a poor prognosis with a prolonged hospital stay, and 18% of mortality in neonates has been caused by late-onset sepsis (5). Early symptoms of neonatal late-onset sepsis are often atypical, and septic shock often occurs due to delayed diagnosis (6). Effective assessment of the risk of sepsis in preterm infants is essential for pediatricians to make early interventions to reduce neonatal mortality.

Increasing evidence indicates that multiple factors affect late-onset sepsis in premature infants. According to the National Institute of Child Health and Human Development, the incidence of infection in premature infants increased with a decrease in birth weight (7). Low levels of thyroid function indexes, such as triiodothyronine (T_3_) and thyroxine (T_4_), have also been reported to be associated with the occurrence of sepsis in premature infants (8, 9).

Previously, Yuejun Huang et al. established a prediction model for late-onset sepsis in premature infants based on predictors, such as birth weight, endotracheal intubation, time of umbilical venous catheterization (UVC), and thyroid function (10). However, the model was constructed based on the data of newborns from Guangdong province, located in the southern part of China, and whether the prediction model was suitable for predicting late-onset sepsis in newborns from other regions in China was still elusive. In addition, the prediction model lacked external data to validate the results.

In the present study, we constructed a prediction model based on the data of premature infants with or without late-onset sepsis in Affiliated Hangzhou First People's Hospital, Zhejiang University School of Medicine, and the data in our study were applied as external validation to evaluate the predictive performance of the prediction model proposed by Yuejun Huang et al.

## Methods

### Study population

The current case-control study collected the data of 397 premature infants recruited from Affiliated Hangzhou First People's Hospital, Zhejiang University School of Medicine between June 2009 and June 2020. The study included infants with a gestational age at birth of <37 weeks who were admitted to the neonatal department within 24 h of birth and stayed in the ward for more than 7 days. Newborns whose mothers had thyroid, liver, kidney, lung, or heart diseases before pregnancy were excluded. Premature infants with congenital malformations or those who were on admission for <7 days were also excluded from the study. All premature infants were randomly divided into the training set (*n* = 278) and the testing set (*n* = 119) at a ratio of 7:3. Informed consent was obtained from the parents of the participants, and this study was approved by the Ethics Committee of Affiliated Hangzhou First People's Hospital, Zhejiang University School of Medicine (No. 202102051224000400219).

### Main variables

The data of main variables of the infants were collected, which included the gender of infants, etiological status (no infection or bacterial infection), endotracheal intubation (yes or no), UVC (yes or no), a peripherally inserted central catheter (yes or no), mechanical ventilation (yes or no), dopamine use (yes or no), albumin use (yes or no), asphyxia (yes or no), antibiotic use (yes or no), thyroid hypofunction (yes or no), birth weight (kg), and gestational age (24 to <28 weeks, 28 to <32 weeks, or 32 to <37 weeks). The clinical data of the mothers were also collected, such as age (years), prenatal glucocorticoid use (yes or no), prenatal antibiotic use (yes or no), premature rupture of fetal membranes (yes or no), the method of delivery (cesarean delivery or vaginal delivery), the season of delivery (spring, summer, autumn, or winter), thyroid stimulating hormone (TSH, μIU/ml), free T_4_ (FT_4_, pmol/L), free T_3_ (FT_3_, pmol/L), T_4_ (nmol/L), and T_3_ (nmol/L).

### Measurement of thyroid function

Thyroid function was measured with a chemiluminescence kit (Beckman Coulter, Prague, Czech Republic) using blood samples of preterm infants taken in the morning between 4 and 7 days after birth. High TSH refers to a TSH >10 mIU/L (11). In addition, T_3_, T_4_, FT_3_, and FT_4_ were corrected using either the 10th percentile or −1 SD as the cut-off value to compensate for the change in TH levels due to gestational age (12, 13). Thyroid hypofunction is defined based on the transient hypothyroxinemia of prematurity (low T_4_ with normal TSH), congenital hypothyroidism (low T_4_ with elevated TSH), low serum T_3_, and high TSH.

### Outcome variables

The outcome was late-onset sepsis in premature infants. The diagnosis of late-onset sepsis in premature infants was based on the Guidelines for the Diagnosis and Treatment of Neonatal Sepsis according to the signs and symptoms as well as laboratory examinations (14). The signs and symptoms, such as unstable temperature, hypotension, poor perfusion with pallor, bradycardia, tachycardia, cyanosis, apnea, lethargy, irritability, seizures, jaundice, abdominal distention, and petechiae, were regarded as suggestive sepsis (15). The blood count, C-reactive protein (CRP), procalcitonin (PCT), and the cultures from blood and other sterile sites were collected to identify bacterial infection. Culture-based diagnostics for late-onset bacterial sepsis was that the infant manifested signs and symptoms of infection after 7 days of age and in line with any of the following: (1) a blood or cerebrospinal fluid (CSF) culture positive for a pathogenic bacterial species, and (2) if the cultures from blood showed conditional pathogenic bacteria (e.g., coagulase-negative staphylococci), the infant was considered to be infected based on the results from other laboratory parameters of infection, such as (1) white blood cell (WBC) count <5 × 10^9^/L or an increased WBC count (age > 3 days, WBC >20 × 10^9^/L); (2) immature neutrophils/total neutrophils ≥0.16; (3) platelet count <100 × 10^9^/L; and (4) C-reactive protein levels ≥8 mg/L (15).

### Statistical analysis

The continuous variables with normal distribution were displayed as mean ± standard deviation (mean ± SD) and comparisons between groups were subjected to the *t*-test. The continuous variables with non-normal distribution were described by M (Q_1_, Q_3_), and the differences between groups were compared by the Wilcoxon rank-sum test. The categorical variables were expressed as *n* (%), and a chi-square test was used for comparing the differences between the groups. Logistic analyses were applied to identify potential predictors, and the prediction model was constructed based on the predictors. After collinearity diagnosis by multivariate logistic regression, predictors with strong collinearity [variance inflation factor (VIF) refers to a relative measure of variance increase caused by the collinearity of independent variables in the estimator of the regression coefficient. VIF > 10 indicates that there is a strong collinearity problem] (16) were excluded in the model. Herein, 70% of the samples were involved as the training set for constructing the model, and 30% of the samples were used as the testing set to test the diagnostic efficiency of the model. The data in our study were applied into the prediction model proposed by Huang et al. (10) as external validation to evaluate its predictive ability. The receiver operator characteristic (ROC) curves and the calibration curves were used to evaluate the differentiation and consistency of the prediction models. The baseline data were analyzed *via* SAS 9.4 statistical analysis software and the ROC curves and the calibration curves were plotted by Python. The value of *p* < 0.05 was considered statistically significant.

## Results

### The baseline characteristics of participants

This study recruited 397 premature infants who were randomly divided into the training set (*n* = 278) and the testing set (*n* = 119). The detailed characteristics of the patients in the training set and the testing set are exhibited in Table 1. In the training set, 138 premature infants were diagnosed to have late-onset sepsis, accounting for 49.64%. In this study, 140 (50.36%) premature infants were boys and 138 (49.64%) were girls. Furthermore, two hundred and eleven infants did not receive endotracheal intubation, accounting for 75.90% of the participants, and 67 received endotracheal intubation, accounting for 24.10% of the participants. Additionally, 188 patients received UVC, accounting for 66.19% of the participants. In total, 103 (37.05%) infants underwent peripheral central catheter insertion. There were 66 (23.74%) patients who received mechanical ventilation. Dopamine was used in 7 (2.52%) patients and albumin was used in 61 (21.94%) patients. Neonatal asphyxia occurred in 85 infants, accounting for 30.58%. In addition, two hundred and forty-six (88.49%) infants received antibiotics. The mean weight of the infants was 1.54 ± 0.41 kg. As for the baseline data of the pregnant mother, the mean age was 31.27 ± 4.86 years old (Table 2).

**Table 1:** The baseline characteristics of participants.

**Variables**	**Total (*n* = 397)**	**Training set (*n* = 278)**	**Testing set (*n* = 119)**
**Infant**			
**Gender**, ***n*** **(%)**			
Boys	212 (53.40)	140 (50.36)	72 (60.50)
Girls	185 (46.60)	138 (49.64)	47 (39.50)
**Etiological result**, ***n*** **(%)**			
No infection	200 (50.38)	140 (50.36)	60 (50.42)
Bacterial infection	197 (49.62)	138 (49.64)	59 (49.58)
**Endotracheal intubation**, ***n*** **(%)**			
No	303 (76.32)	211 (75.90)	92 (77.31)
Yes	94 (23.68)	67 (24.10)	27 (22.69)
**Umbilical vein catheterization**, ***n*** **(%)**			
No	259 (65.24)	184 (66.19)	75 (63.03)
Yes	138 (34.76)	94 (33.81)	44 (36.97)
**Peripherally inserted central catheter**, ***n*** **(%)**			
No	246 (61.96)	175 (62.95)	71 (59.66)
Yes	151 (38.04)	103 (37.05)	48 (40.34)
**Mechanical ventilation**, ***n*** **(%)**			
No	303 (76.32)	212 (76.26)	91 (76.47)
Yes	94 (23.68)	66 (23.74)	28 (25.53)
**Dopamine use**, ***n*** **(%)**			
No	389 (97.98)	271 (97.48)	118 (99.16)
Yes	8 (2.02)	7 (2.52)	1 (0.84)
**Albumin use**, ***n*** **(%)**			
No	297 (74.81)	217 (78.06)	80 (67.23)
Yes	100 (25.19)	61 (21.94)	39 (32.77)
**Asphyxia**, ***n*** **(%)**			
No	268 (67.51)	193 (69.42)	75 (63.03)
Yes	129 (32.49)	85 (30.58)	44 (36.97)
**Antibiotic use**, ***n*** **(%)**			
No	50 (12.59)	32 (11.51)	18 (15.13)
Yes	347 (87.41)	246 (88.49)	101 (84.87)
Birth Weight (kg), Mean ± SD	1.54 ± 0.41	1.54 ± 0.41	1.51 ± 0.43
**Mother**			
Age (years), Mean ± SD	31.34 ± 4.96	31.27 ± 4.86	31.50 ± 5.20
**Gestational age (W)**, ***n*** **(%)**			
24- <28	58 (14.61)	40 (14.39)	18 (15.13)
28- <32	245 (61.71)	170 (61.15)	75 (63.03)
2- <37	94 (23.68)	68 (24.46)	26 (21.85)
**Prenatal glucocorticoid use**, ***n*** **(%)**			
No	62 (15.62)	44 (15.83)	18 (15.13)
Yes	335 (84.38)	234 (84.17)	101 (84.87)
**Prenatal antibiotic use**, ***n*** **(%)**			
No	202 (50.88)	143 (51.44)	59 (49.58)
Yes	195 (49.12)	135 (48.56)	60 (50.42)
**Premature rupture of fetal membranes**, ***n*** **(%)**			
No	217 (68.26)	190 (68.35)	81 (68.07)
Yes	126 (31.74)	88 (31.65)	38 (31.93)
**Method of delivery**, ***n*** **(%)**			
Cesarean delivery	309 (77.83)	216 (77.70)	93 (78.15)
Vaginal delivery	88 (22.17)	62 (22.30)	26 (21.85)
**Season of delivery**, ***n*** **(%)**			
Spring	89 (22.42)	69 (24.82)	20 (16.81)
Summer	139 (35.01)	92 (33.09)	47 (39.50)
Autumn	82 (20.65)	52 (18.71)	30 (25.21)
Winter	87 (21.91)	65 (23.38)	22 (18.49)
**Thyroid hypofunction**			
No	226 (56.93)	155 (55.76)	71 (59.66)
Yes	171 (43.07)	123 (44.24)	48 (40.34)

**Table 2:** Comparisons of characteristics between the control group and the case group in the training set.

**Variables**	**Total (*n* = 278)**	**Control Group (*n* = 140)**	**Case Group (*n* = 138)**	**Statistic**	***P***
**Infant**					
Gender, *n* (%)				χ^2^ = 0.361	0.548
Male	140 (50.36)	68 (48.57)	72 (52.17)		
Female	138 (49.64)	72 (51.43)	66 (47.83)		
**Etiological result**, ***n*** **(%)**				χ^2^ = 278.000	<0.001
No infection	140 (50.36)	140 (100.00)	0 (0.00)		
Bacterial infection	138 (49.64)	0 (0.00)	138 (100.00)		
**Endotracheal intubation**, ***n*** **(%)**				χ^2^ = 48.151	<0.001
No	211 (75.90)	131 (93.57)	80 (57.97)		
Yes	67 (24.10)	9 (6.43)	58 (42.03)		
**Umbilical vein catheterization**, ***n*** **(%)**				χ^2^ = 2.853	0.091
No	184 (66.19)	86 (61.43)	98 (71.01)		
Yes	94 (33.81)	54 (38.57)	40 (28.99)		
**Peripherally inserted central catheter**, ***n*** **(%)**				χ^2^ = 2.912	0.088
No	175 (62.95)	95 (67.86)	80 (57.97)		
Yes	103 (37.05)	45 (32.14)	58 (42.03)		
**Mechanical ventilation**, ***n*** **(%)**				χ^2^ = 46.690	<0.001
No	212 (76.26)	131 (93.57)	81 (58.70)		
Yes	66 (23.74)	9 (6.43)	57 (41.30)		
**Dopamine use**, ***n*** **(%)**				-	0.280
No	271 (97.48)	138 (98.57)	133 (96.38)		
Yes	7 (2.52)	2 (1.43)	5 (3.62)		
**Albumin use**, ***n*** **(%)**				χ^2^ = 3.793	0.051
No	217 (78.06)	116 (82.86)	101 (73.19)		
Yes	61 (21.94)	24 (17.14)	37 (26.81)		
**Asphyxia**, ***n*** **(%)**				χ^2^ = 12.920	<0.001
No	193 (69.42)	111 (79.29)	82 (59.42)		
Yes	85 (30.58)	29 (20.71)	56 (40.58)		
**Antibiotic use**, ***n*** **(%)**				χ^2^ = 8.783	0.003
No	32 (11.51)	24 (17.14)	8 (5.80)		
Yes	246 (88.49)	116 (82.86)	130 (94.20)		
Birth weight (kg), Mean ± SD	1.54 ± 0.41	1.63 ± 0.38	1.45 ± 0.42	*t* = 3.78	<0.001
**Mother**					
Age (years), Mean ± SD	31.27 ± 4.86	31.65 ± 5.06	30.88 ± 4.65	*t* = 1.31	0.190
**Gestational age (W)**, ***n*** **(%)**				χ^2^ = 9.363	0.009
24- <28	40 (14.39)	12 (8.57)	28 (20.29)		
28- <32	170 (61.15)	87 (62.14)	83 (60.14)		
32- <37	68 (24.46)	41 (29.29)	27 (19.57)		
**Prenatal glucocorticoid use**, ***n*** **(%)**				χ^2^ = 2.874	0.090
No	44 (15.83)	17 (12.14)	27 (19.57)		
Yes	234 (84.17)	123 (87.86)	111 (80.43)		
Prenatal antibiotic use, *n* (%)				χ^2^ = 2.084	0.149
No	143 (51.44)	66 (47.14)	77 (55.80)		
Yes	135 (48.56)	74 (52.86)	61 (44.20)		
Premature rupture of fetal membranes, *n* (%)				χ^2^ = 0.188	0.664
No	190 (68.35)	94 (67.14)	96 (69.57)		
Yes	88 (31.65)	46 (32.86)	42 (30.43)		
**Method of delivery**, ***n*** **(%)**				χ^2^ = 0.124	0.725
Cesarean delivery	216 (77.70)	110 (78.57)	106 (76.81)		
Vaginal delivery	62 (22.30)	30 (21.43)	32 (23.19)		
**Season of delivery*****, n*** **(%)**				χ^2^ = 4.675	0.197
Spring	69 (24.82)	34 (24.29)	35 (25.36)		
Summer	92 (33.09)	47 (33.57)	45 (32.61)		
Autumn	52 (18.71)	32 (22.86)	20 (14.49)		
Winter	65 (23.38)	27 (19.29)	38 (27.54)		
**Thyroid hypofunction**, ***n*** **(%)**				χ^2^ = 0.907	0.341
No	155 (55.76)	82 (58.57)	73 (52.90)		
Yes	123 (44.24)	58 (41.43)	65 (47.10)		

### Univariate analysis of factors affecting the risk of late-onset sepsis in premature infants

As exhibited in Table 3, compared with patients without endotracheal intubation, the risk of late-onset sepsis in premature infants was 10.553-fold higher in patients with endotracheal intubation [odds ratio (*OR*) = 10.553, 95% confidence interval (*CI*): 4.959–22.458]. Mechanical ventilation treatment increased the risk of late-onset sepsis in premature infants (*OR* = 10.243, 95% *CI*: 4.811–21.806). Asphyxia increased the risk of late-onset sepsis by 2.614-fold when compared with infants without asphyxia (*OR* = 2.614, 95% *CI*: 1.536–4.447). Relative to infants without antibiotics use, the risk of late-onset sepsis was increased by 3.362-fold in those with antibiotic use (*OR* = 3.362, 95% *CI*: 1.454–7.775). Increased birth weight of infants was associated with a decreased risk of late-onset sepsis (*OR* = 0.312, 95% *CI*: 0.165–0.588). Compared with infants delivered at a gestational age of 24 to <28 weeks, the risk of late-onset sepsis decreased by 0.409-fold in infants delivered at a gestational age of 28 to <32 weeks (*OR*) and further by 0.282-fold in infants delivered at a gestational age of 32 to <37 weeks (*OR* = 0.282, 95% *CI*: 0.123–0.649).

**Table 3:** The potential predictors for the risk of late-onset sepsis in premature infants.

**Variables**	**β**	**Standardized Coefficients**	**OR (95%CI)**	***P*-value**
**Infant**				
**Gender of infants**				
Boys	Ref			
Girls	−0.144	−0.040	0.866 (0.541, 1.386)	0.548
**Endotracheal intubation**				
No	Ref			
Yes	2.356	0.557	10.553 (4.959, 22.458)	<0.001
**Umbilical vein catheterization**				
No	Ref			
Yes	−0.431	−0.113	0.650 (0.394, 1.073)	0.092
**Peripherally inserted central catheter**				
No	Ref			
Yes	0.426	0.114	1.531 (0.938, 2.498)	0.089
**Mechanical ventilation**				
No	Ref			
Yes	2.327	0.547	10.243 (4.811, 21.806)	<0.001
**Dopamine use**				
No	Ref			
Yes	0.953	0.082	2.592 (0.494, 13.589)	0.260
**Albumin use**				
No	Ref			
Yes	0.571	0.131	1.771 (0.993, 3.159)	0.053
**Asphyxia**				
No	Ref			
Yes	0.961	0.245	2.614 (1.536, 4.447)	<0.001
**Antibiotic use**				
No	Ref			
Yes	1.213	0.214	3.362 (1.454, 7.775)	0.005
**Thyroid hypofunction**				
No	Ref			
Yes	0.230	0.242	1.261 (0.784, 2.022)	0.342
Birth Weight	−1.165	−0.261	0.312 (0.165, 0.588)	<0.001
**Mother**				
Age	−0.033	−0.088	0.968 (0.922, 1.016)	0.190
**Gestational age (W**)				
24- <28	Ref			
28- <32	−0.894	−0.201	0.409 (0.195, 0.857)	0.018
32- <37	−1.265	−0.300	0.282 (0.123, 0.649)	0.003
**Prenatal glucocorticoid use**				
No	Ref			
Yes	−0.565	−0.114	0.568 (0.294, 1.098)	0.093
**Prenatal antibiotic use**				
No	Ref			
Yes	−0.347	−0.096	0.707 (0.441, 1.133)	0.149
**Premature rupture of fetal membranes**				
No	Ref			
Yes	−0.112	−0.029	0.894 (0.539, 1.483)	0.664
**Method of delivery**				
Cesarean delivery	Ref			
Vaginal delivery	0.102	0.023	1.107 (0.629, 1.948)	0.725
**Season of delivery**				
Spring	Ref			
Summer	−0.073	−0.019	0.930 (0.498, 1.737)	0.820
Autumn	−0.500	−0.108	0.607 (0.292, 1.262)	0.181
Winter	0.313	0.073	1.367 (0.691, 2.706)	0.369

### Construction of the prediction model

The prediction model for late-onset sepsis in premature infants was constructed based on the predictors identified *via* univariate analysis. The results of multicollinearity analysis revealed that endotracheal intubation (VIF = 54.49320) and mechanical ventilation (VIF = 53.36468) indicated that they had strong collinearity with other variables (Supplementary Table 1). Finally, the prediction model was ln ($\frac{P}{1-P}$)= 4.927 + 0.346 (Male) – 0.299 (28w-32w gestational age) – 0.871 (32w−37w gestational age) + 0.974 (antibiotic use) + 0.698 (asphyxia) – 1.894 (birth weight) + 0.266 (albumin use) – 1.586 (prenatal glucocorticoid use) – 1.217 (umbilical vein catheterization) + 0.135 (premature rupture of fetal membranes) – 0.410 (peripherally inserted central catheter) – 0.027 (age of mother) + 0.368 (dopamine use) + 0.356 Summer + 0.524 Spring + 0.978 Winter (Table 4).

**Table 4:** Construction of the prediction model.

**Coefficients**	**β**	**Standardized Coefficients**	**OR (95%CI)**	***P*-value**
Intercept	4.927	1.473		<0.001
Gender of infant (Male)	0.346	0.290	1.41 (0.80–2.49)	0.233
**Gestational age**				
28- <32	−0.299	0.488	0.74 (0.29–1.93)	0.540
32- <37	−0.871	0.649	0.42 (0.12-1.49)	0.180
Antibiotic use (Yes)	0.974	0.497	2.65 (1.01–7.01)	0.050
Asphyxia (Yes)	0.698	0.324	2.01 (1.07–3.79)	0.031
Birth weight	−1.894	0.523	0.15 (0.05–0.42)	<0.001
Albumin use (Yes)	0.266	0.352	1.31 (0.65–2.60)	0.450
Prenatal glucocorticoid use (Yes)	−1.586	0.464	0.20 (0.08–0.51)	<0.001
Umbilical vein catheterization (Yes)	−1.217	0.345	0.30 (0.15–0.58)	<0.001
Premature rupture of fetal membranes (Yes)	0.135	0.305	1.14 (0.63–2.08)	0.658
Peripherally inserted central catheter (Yes)	−0.410	0.360	0.66 (0.33–1.35)	0.256
Age of mother	−0.027	0.029	0.97 (0.92–1.03)	0.337
Dopamine use (Yes)	0.368	0.928	1.45 (0.23–8.90)	0.692
**Season of delivery**				
Summer	0.356	0.398	1.43 (0.65–3.12)	0.371
Spring	0.356	0.398	1.43 (0.65–3.12)	0.371
Winter	0.978	0.428	2.66 (1.15–6.16)	0.022

### Validation of the prediction model

**Figure 1:**
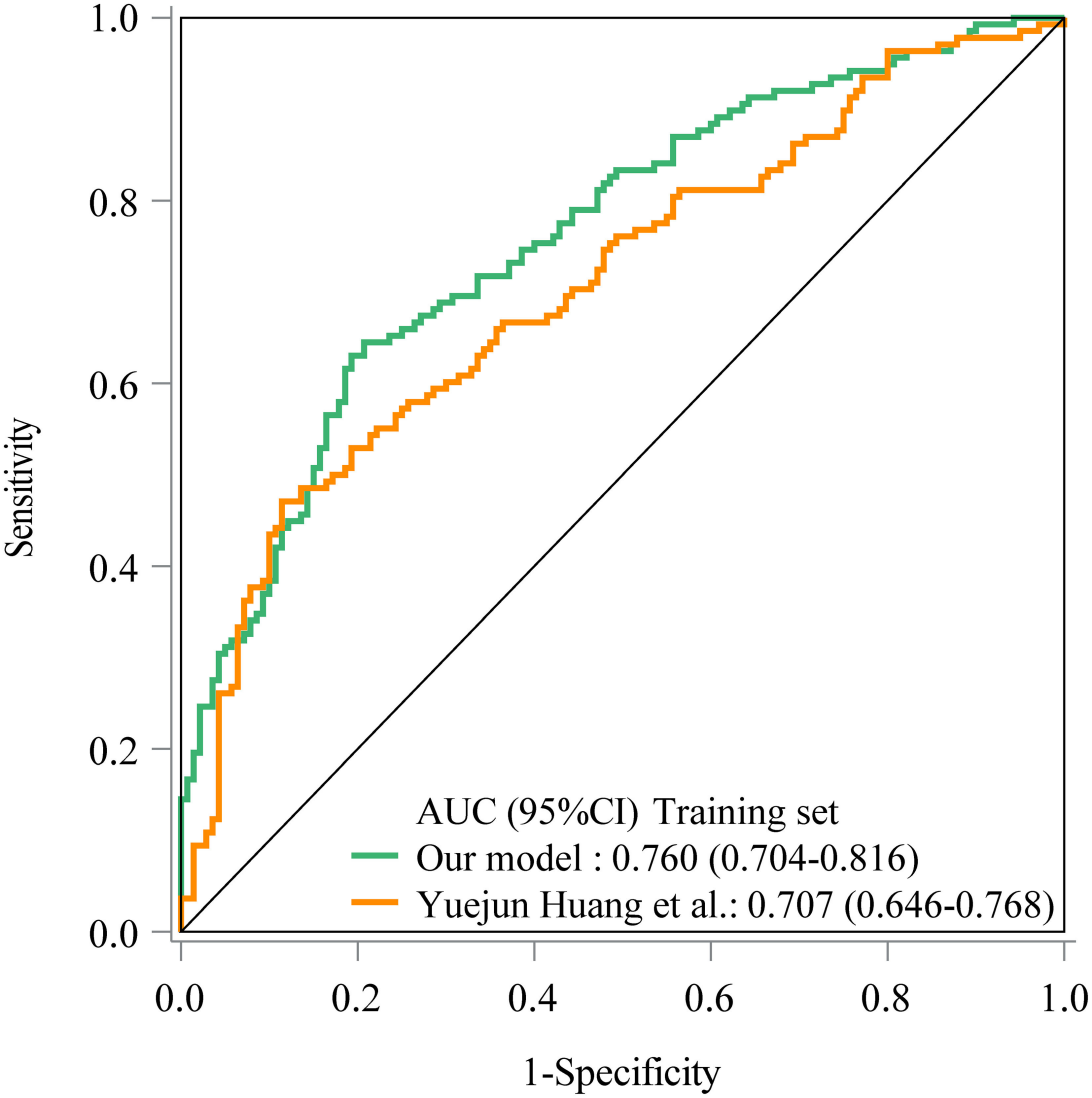
The receiver operator characteristic (ROC) curve showed the predictive ability of the prediction model in the training set in our model and Yuejun Huang et al.'s model.

**Figure 2:**
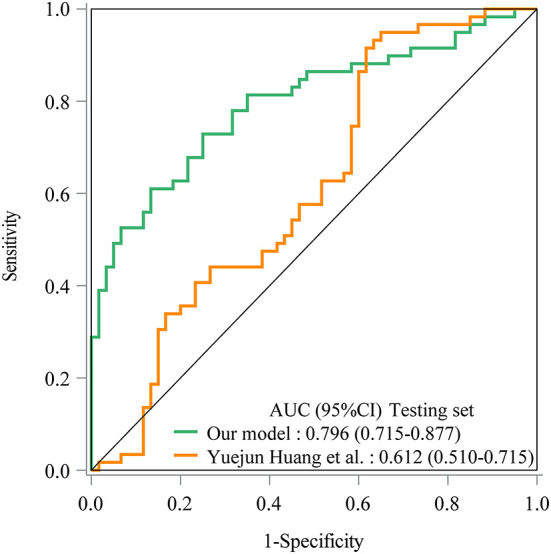
The ROC curve showed the predictive ability of the prediction model in the testing set in our model and Yuejun Huang et al.'s model.

**Figure 3:**
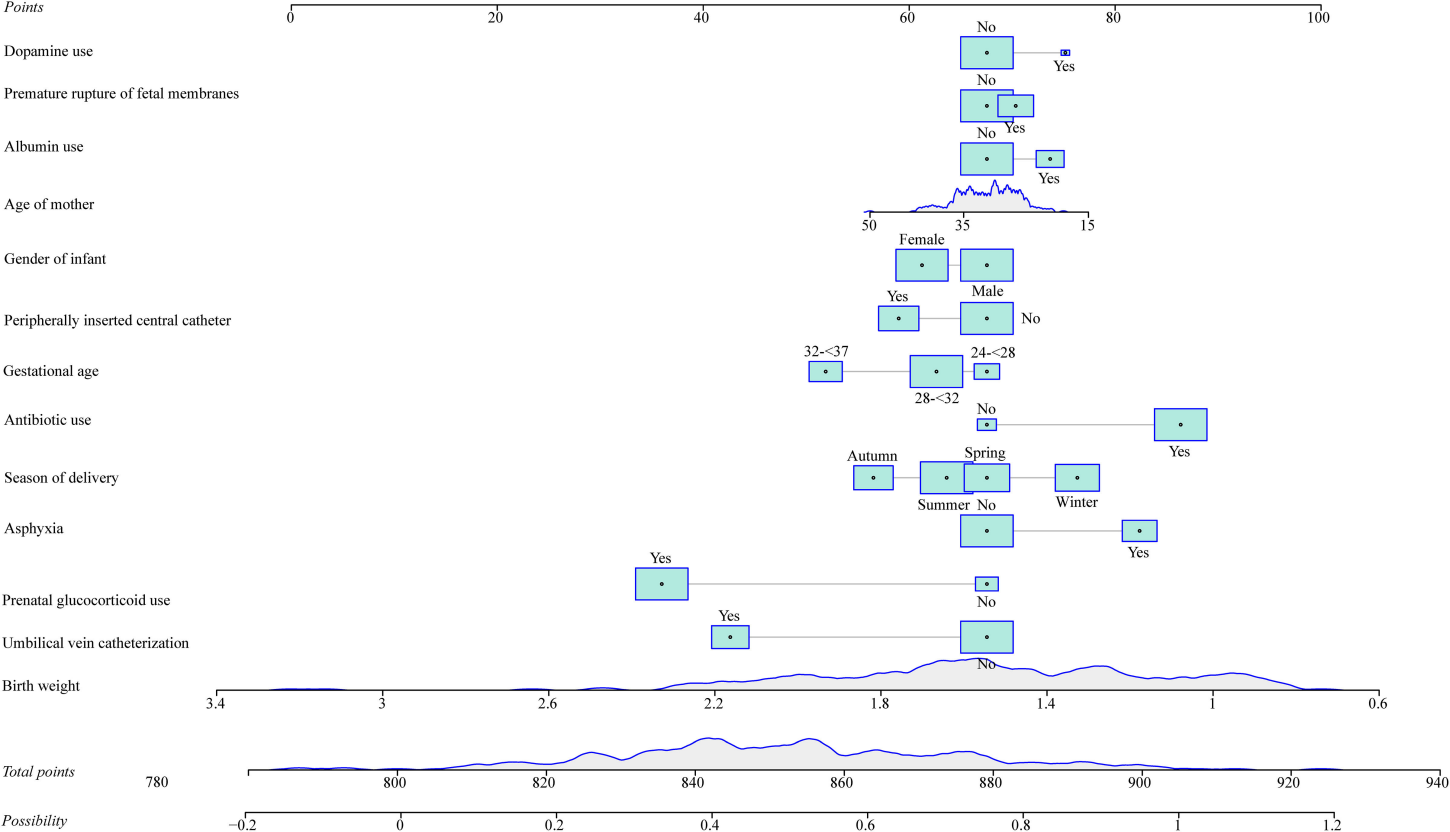
The nomogram was plotted based on the prediction model.

**Figure 4:**
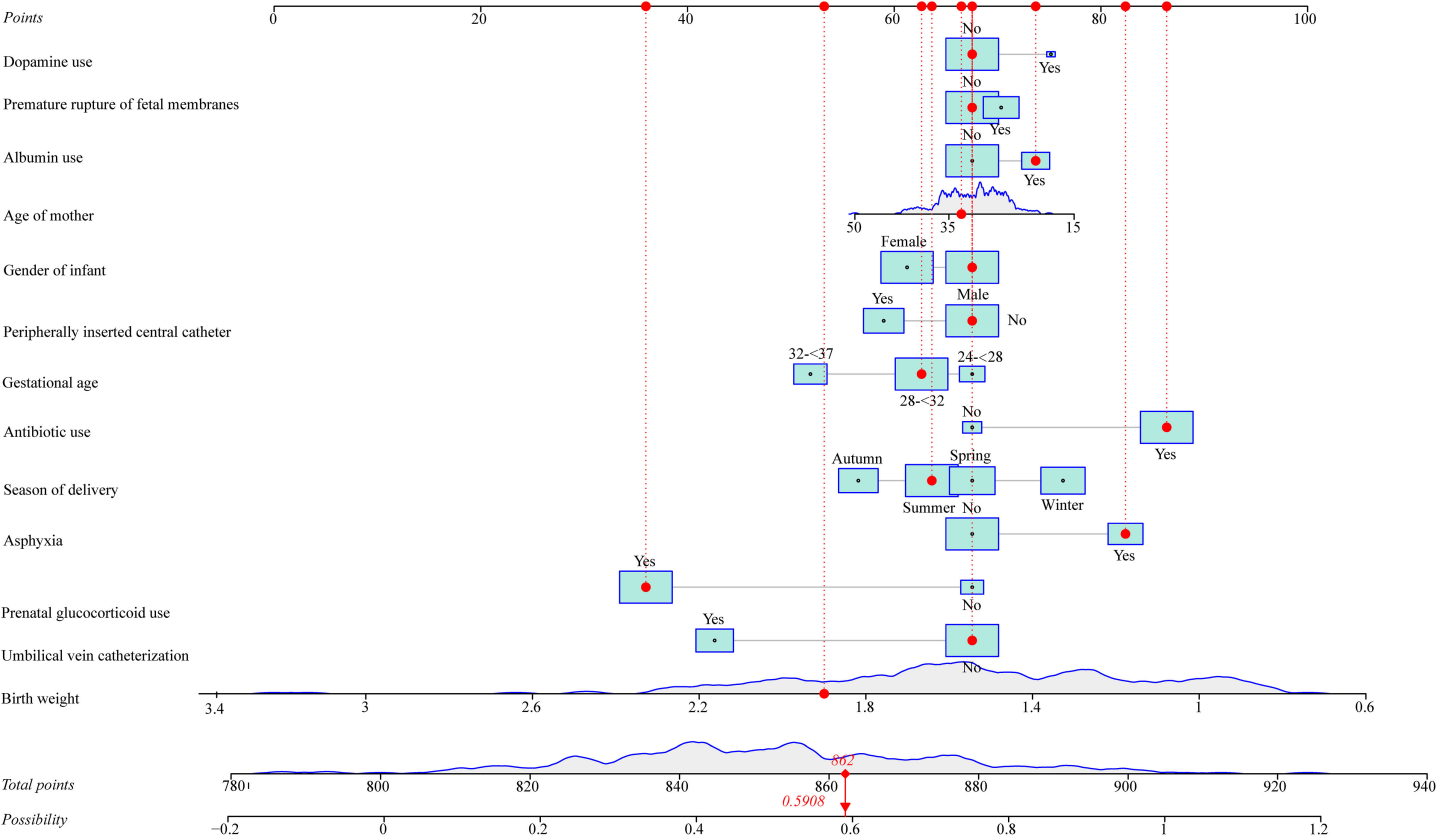
A sample was randomly selected from the training set to validate the performance of the prediction model.

As seen in Figure 1, in the training set, the AUC was 0.760 (95% *CI*: 0.704–0.816), the accuracy was 0.719 (0.663–0.771), the sensitivity was 0.645 (0.565–0.725), the specificity was 0.793 (0.726–0.860), the negative predictive value (NPV) was 0.694 (0.622–0.765), and the positive predictive value (PPV) was 0.754 (0.677–0.832) (Table 5). In the testing set, the AUC was 0.796 (95% *CI*: 0.715–0.877) (Figure 2). The cutoff value was 0.539. The accuracy was 0.714 (95% *CI*: 0.624–0.793), the sensitivity was 0.678 (95% *CI*: 0.559–0.793), the specificity was 0.750 (95% *CI*: 0.640–0.860), the NPV was 0.703 (95% *CI*: 0.591–0.815), and the PPV was 0.727 (95% *CI*: 0.610–0.845) (Table 5). The nomogram was plotted based on the prediction model (Figure 3). A sample was randomly selected from the training set to validate the performance of the prediction model. The baseline data of the sample were a male infant with a gestational age at 24 to <28 weeks, the use of albumin, and asphyxia. The birth weight of the infant was 1.9 kg. The infant did not use UVC, peripheral catheterization into the central vein, or dopamine and was born in summer. The mother was 33 years old and used prenatal glucocorticoid. The total score of this infant was 862, and the possibility of late-onset sepsis was 0.5908 (Figure 4). The predicted result was similar to the actual result, and the infant had late-onset sepsis, indicating that the predictive ability of the model was good.

**Table 5:** The predictive value of the prediction model.

**Character (95%CI)**	**Training set**	**Testing set**
**Our model**		
AUC	0.760 (0.704–0.816)	0.796 (0.715–0.877)
Accuracy	0.719 (0.663–0.771)	0.714 (0.624–0.793)
Sensitivity	0.645 (0.565–0.725)	0.678 (0.559–0.797)
Specificity	0.793 (0.726–0.860)	0.750 (0.640–0.860)
NPV	0.694 (0.622–0.765)	0.703 (0.591–0.815)
PPV	0.754 (0.677–0.832)	0.727 (0.610–0.845)
Cutoff	0.539	0.539
**Model by Huang et al**. **(****10****)**		
AUC	0.707 (0.646–0.768)	0.612 (0.510–0.715)
Accuracy	0.680 (0.622–0.734)	0.580 (0.486–0.670)
Sensitivity	0.471 (0.386–0.558)	0.441 (0.312–0.576)
Specificity	0.886 (0.821–0.933)	0.717 (0.586–0.825)
NPV	0.629 (0.558–0.697)	0.566 (0.447–0.679)
PPV	0.802 (0.699–0.883)	0.605 (0.444–0.750)
Cutoff	0.164	0.164
*P*	0.210	0.006

AUC, area under the curve; NPV, negative predictive value; PPV, positive predictive value.

**Figure 5:**
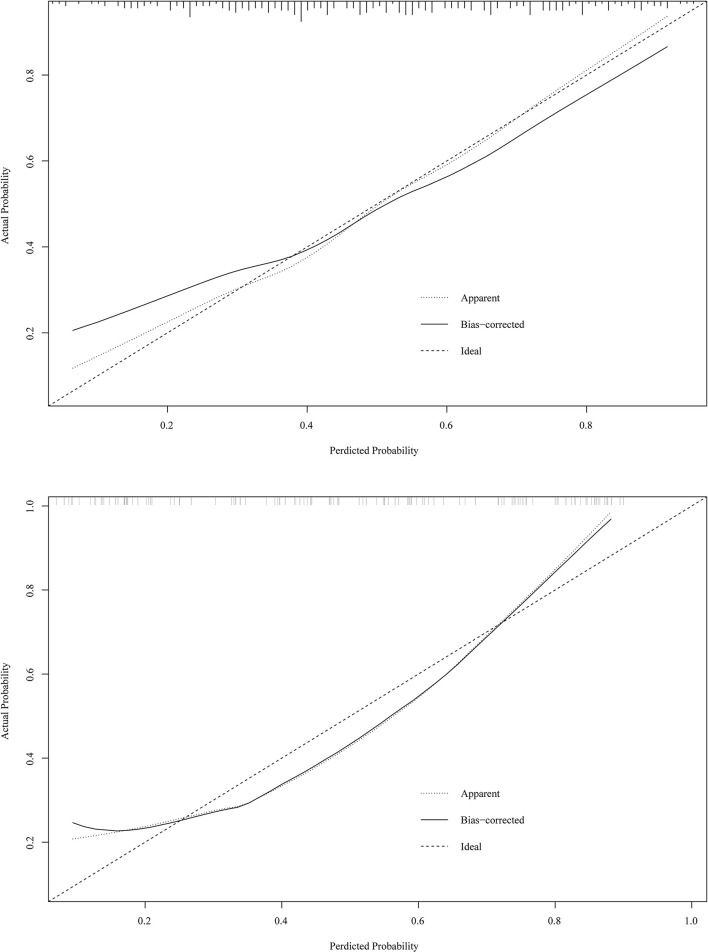
The calibration curves of the training set and the testing set were respectively plotted.

The prediction model from Huang et al. (10) was applied in our data. The prediction model of Yuejun Huang et al. was ln ($\frac{P}{1-P}$) = −1.995 (birth weight) + 0.297 (umbilical vein catheterization) + 1.648 (endotracheal intubation) + 1.407 (thyroid hypofunction) + 0.024 (age of mother) + 0.029 (delivery mode) - 0.143 (gestational age) + 0.053 asphyxia – 0.033 (peripherally inserted central catheter) + 0.037 (mechanical ventilation). In the training set, the AUC was 0.707 (95% *CI*: 0.646–0.768) (Figure 1), the accuracy was 0.680 (95% *CI*: 0.622–0.734), the sensitivity was 0.471 (0.386–0.558), the specificity was 0.886 (95% *CI*: 0.821–0.933), the NPV was 0.629 (95% *CI*: 0.558–0.697), and the PPV was 0.802 (95% *CI*: 0.699–0.883). In the testing set, the AUC was 0.612 (95% *CI*: 0.510–0.715) (Figure 2), the accuracy was 0.580 (95% *CI*: 0.486–0.670), the sensitivity was 0.441 (95% *CI*: 0.312–0.576), the specificity was 0.717 (95% *CI*: 0.586–0.825), the NPV was 0.566 (95% *CI*: 0.447–0.679), and the PPV was 0.605 (95% *CI*: 0.444–0.750) in the testing set (Table 5). The AUC in the testing set of our model was statistically higher than the AUC in the testing set using the model by Huang et al. (10) (*p* = 0.006). The calibration curves of the training set and the testing set were respectively plotted, and it revealed that our prediction model had a better predictive value (Figure 5).

## Discussion

In the present study, the data of 397 premature infants were analyzed to identify the predictors of late-onset sepsis in those patients and established a prediction model for late-onset sepsis in premature infants. The results delineated that endotracheal intubation, mechanical ventilation treatment, neonatal asphyxia, antibiotics use, birth weight, and gestational age were predictors associated with the risk of late-onset sepsis in premature infants. The prediction model was constructed based on predictors, and the predictive performance was good in the training set and the testing set. Additionally, our data were applied to the prediction model from Huang et al. (10), and the results depicted that the predictive ability of our prediction model might be better than the prediction model from Yuejun Huang et al. (10).

Endotracheal intubation is a vital intervention for the stabilization of critically ill infants (17). As an invasive medical operation, endotracheal intubation can cause damage to the upper respiratory mucosa and destroy the natural immune barrier of the body (18). Prolonged endotracheal intubation can also change the bacterial colonization of the respiratory tract, which might increase the incidence of sepsis (19). This supported the results of our study, which revealed that the use of endotracheal intubation was an independent risk factor for late-onset sepsis in preterm infants. According to the study by Manandhar S et al., each additional day of mechanical ventilation application increased the risk of sepsis by 0.086 times for patients in a neonatal intensive care unit (20). The association between mechanical ventilation use and the risk of late-onset sepsis was also identified by Yapicioglu et al. (21). These studies gave support to the findings in the current study. Infants with asphyxia also had a higher risk of late-onset sepsis in the current study. Perinatal asphyxia is one of the main causes of neonatal deaths all through the world (22). Perinatal asphyxia might result in the disorder of thyroid function and might lead to low levels of thyroid hormones in newborns, and thyroid hormones are essential participants in regulating the innate immune system (9, 23). Low thyroid hormones levels were reported to increase the risk of bacterial sepsis and associated with the poor prognosis of newborns and young children (24, 25). Antibiotics are the most commonly applied treatment for suspected sepsis in neonatal intensive care units because of the diagnostic challenges of sepsis and the relative immunosuppression of newborns (26). The use of antimicrobial agents, such as antibiotics, is reported to be a risk factor for multidrug-resistant bacterial infections, which may lead to sepsis (27). This provided evidence to the results of this study, showing that the use of antibiotics was a risk factor for late-onset sepsis in preterm infants. This serves to remind the pediatricians to use antibiotics with caution in preterm infants. According to a report from the Turkish Nosocomial Infections Study Group, the frequency of sepsis was increased by 22% in infants with a birth weight of <1,500 g, 6% in infants with a birth weight of 1,500–2,500 g, and 3% in infants with a birth weight of ≥2,500 g, demonstrating that the risk of sepsis was increased with the decrease in birth weight of the newborns (28). This provided evidence to the findings in the present study, uncovering that increased birth weight of infants was associated with a decreased risk of late-onset sepsis. A systematic review and meta-analysis conducted in India analyzed risk factors for neonatal sepsis and found that neonates with a gestational age of <37 weeks have a higher risk of sepsis (29). Similarly, we found that there might be a negative association between the risk of late-onset sepsis and the gestation age in preterm infants. This suggests that preterm infants with low birth weight and small for gestation age should receive timely interventions to prevent the occurrence of late-onset sepsis.

In this study, factors associated with the risk of late-onset sepsis in preterm patients were assessed, and a prediction model was constructed based on predictors in the training set. The validation was conducted in the testing set. ROC curves were drawn to present the diagnostic ability of the prediction model. The AUC was 0.760 in the training set and 0.796 in the testing set, implying the good predictive performance of the model. A nomogram for predicting the risk of late-onset sepsis in preterm patients was also plotted, which allowed us to easily calculate the score directly from the graph and obtain the possibility of late-onset sepsis in preterm patients. An actual case in the training set was randomly chosen to validate the predictive ability of our model, and the predicted results were similar to the actual outcome of the case. Moreover, the external validation of the previous prediction model for late-onset sepsis in preterm patients proposed by Huang et al. (10) was performed using our data, and it showed that the AUC was 0.707 in the training set and 0.612 in the testing set. All these findings indicated that the prediction model in our study had a good predictive value for late-onset sepsis in preterm patients, which might help identify patients who have a high risk of late-onset sepsis and provide timely intervention to prevent the occurrence of late-onset sepsis in those patients. The nomogram was platted, which we believe is easy for pediatricians to use to calculate the possibility of infants having late-onset sepsis. The predictors were common information and easy to be collected clinically. The prediction model can guide evaluation and treatment decisions.

Several limitations exist in the current study. First, the sample size was small, which might have decreased the statistical power of our results. Second, the prediction model lacks external validation in other data. Third, important variables, such as the status of inflammation in the chorionic plate, were not included in this study. In the future, more well-designed studies with larger sample sizes or/and different populations are required to verify the findings of our study.

## Conclusions

The predictors of late-onset sepsis in preterm infants were analyzed, and a prediction model was established based on the predictors, which presented a good predictive ability. The findings of our study might offer a reference to identify preterm infants with a high risk of late-onset sepsis and provide timely treatments to reduce the occurrence of late-onset sepsis in those patients.

## Data availability statement

The raw data supporting the conclusions of this article will be made available by the authors, without undue reservation.

## Ethics statement

The studies involving human participants were reviewed and approved by Affiliated Hangzhou First People's Hospital, Zhejiang University School of Medicine (No. 202102051224000400219). Written informed consent to participate in this study was provided by the participants' legal guardian/next of kin.

## Author contributions

XS: designed the study and wrote the manuscript. XS, XL, and YW: collected, analyzed, and interpreted the data. XL and YW: critically reviewed, edited, and approved the manuscript. All authors read and approved the final manuscript.

## Conflict of interest

The authors declare that the research was conducted in the absence of any commercial or financial relationships that could be construed as a potential conflict of interest.

## Publisher's note

All claims expressed in this article are solely those of the authors and do not necessarily represent those of their affiliated organizations, or those of the publisher, the editors and the reviewers. Any product that may be evaluated in this article, or claim that may be made by its manufacturer, is not guaranteed or endorsed by the publisher.
