# Supplementary material for: Prediction for late-onset sepsis in preterm infants based on data from East China
Source: Front Pediatr. 2022 Sep 14;10:924014. doi: 10.3389/fped.2022.924014 (PMC9515484; doi:10.3389/fped.2022.924014)
Supplement: Supplementary file 1 [file Table_1.docx]

Supplementary Table 1 The results of multicollinearity analysis

| **Variables** | **β** | **S.E** | **t** | ***P*** | **TOL** | **VIF** |
| --- | --- | --- | --- | --- | --- | --- |
| Gender | -0.04234 | 0.05661 | -0.75 | 0.4552 | 0.88601 | 1.12866 |
| Gestational age | -0.06388 | 0.06008 | -1.06 | 0.2887 | 0.51969 | 1.92421 |
| Age of mother | -0.00367 | 0.00569 | -0.65 | 0.5192 | 0.92973 | 1.07558 |
| Endotracheal intubation | 0.43121 | 0.45981 | 0.94 | 0.3492 | **0.01835** | **54.49320** |
| Mechanical ventilation | -0.05792 | 0.45737 | -0.13 | 0.8993 | **0.01874** | **53.36468** |
| Asphyxia | 0.01571 | 0.06876 | 0.23 | 0.8194 | 0.70710 | 1.41423 |
| Antibiotic use | 0.15605 | 0.08932 | 1.75 | 0.0818 | 0.87333 | 1.14504 |
| Birth weight | -0.30903 | 0.09976 | -3.10 | 0.0022 | 0.43506 | 2.29855 |
| Albumin use | 0.03101 | 0.07101 | 0.44 | 0.6627 | 0.82172 | 1.21696 |
| Prenatal glucocorticoid use | -0.24227 | 0.08135 | -2.98 | 0.0032 | 0.80490 | 1.24240 |
| Umbilical vein catheterization | -0.19339 | 0.06584 | -2.94 | 0.0036 | 0.73158 | 1.36691 |
| Premature rupture of fetal membranes | 0.03331 | 0.06060 | 0.55 | 0.5831 | 0.89318 | 1.11959 |
| Peripherally inserted central catheter | -0.07580 | 0.06922 | -1.10 | 0.2745 | 0.63511 | 1.57454 |
| Dopamine use | -0.00878 | 0.17424 | -0.05 | 0.9598 | 0.95234 | 1.05005 |
| Season of birth | 0.02782 | 0.02472 | 1.13 | 0.2614 | 0.96376 | 1.03760 |

TOL: tolerance, VIF: variance inflation factor
